# Effect of screening and management of diabetes during pregnancy on stillbirths

**DOI:** 10.1186/1471-2458-11-S3-S2

**Published:** 2011-04-13

**Authors:** Madiha Syed, Hasan Javed, Mohammad Yawar Yakoob, Zulfiqar A Bhutta

**Affiliations:** 1 Division of Women & Child Health, The Aga Khan University, Stadium Road, P.O. Box 3500, Karachi, Pakistan

## Abstract

**Background:**

Diabetes during pregnancy is associated with significant risk of complications to the mother, fetus and newborn. We reviewed the potential impact of early detection and control of diabetes mellitus during pregnancy on stillbirths for possible inclusion in the Lives Saved Tool (LiST).

**Methods:**

A systematic literature search up to July 2010 was done to identify all published randomized controlled trials and observational studies. A standardized data abstraction sheet was employed and data were abstracted by two independent authors. Meta-analyses were performed with different sub-group analyses. The analyses were graded according to the CHERG rules using the adapted GRADE criteria and recommendations made after assessing the overall quality of the studies included in the meta-analyses.

**Results:**

A total of 70 studies were selected for data extraction including fourteen intervention studies and fifty six observational studies. No randomized controlled trials were identified evaluating early detection of diabetes mellitus in pregnancy versus standard screening (glucose challenge test between 24^th^ to 28^th^ week of gestation) in pregnancy. Intensive management of gestational diabetes (including specialized dietary advice, increased monitoring and tailored dietary therapy) during pregnancy (3 studies: 3791 participants) versus conventional management (dietary advice and insulin as required) was associated with a non-significant reduction in the risk of stillbirths (RR 0.20; 95% CI: 0.03-1.10) (‘moderate’ quality evidence). Optimal control of serum blood glucose versus sub-optimal control was associated with a significant reduction in the risk of perinatal mortality (2 studies, 5286 participants: RR=0.40, 95% CI 0.25- 0.63), but not stillbirths (3 studies, 2469 participants: RR=0.51, 95% CI 0.14-1.88). Preconception care of diabetes (information about need for optimization of glycemic control before pregnancy, assessment of diabetes complications, review of dietary habits, intensification of capillary blood glucose self-monitoring and optimization of insulin therapy) versus none (3 studies: 910 participants) was associated with a reduction in perinatal mortality (RR=0.29, 95% CI 0.14 -0.60). Using the Delphi process for estimating effect size of optimal diabetes recognition and management yielded a median effect size of 10% reduction in stillbirths.

**Conclusions:**

Diabetes, especially pre-gestational diabetes with its attendant vascular complications, is a significant risk factor for stillbirth and perinatal death. Our review highlights the fact that very few studies of adequate quality are available that can provide estimates of the effect of screening for aid management of diabetes in pregnancy on stillbirth risk. Using the Delphi process we recommend a conservative 10% reduction in the risk of stillbirths, as a point estimate for inclusion in the LiST.

## Background

With increasing prevalence of diabetes, the prevalence of diabetes complicated pregnancies is also increasing. Normally, as the pregnancy progresses, mothers experience lower glucose levels compared to the non pregnant state. With the progression of pregnancy there is lowered glucose tolerance, increasing glucose and insulin levels. Although this is a normal physiological process, it can become pathological in 3-6 % pregnancies [1]. Gestational diabetes is defined as any degree of glucose intolerance with onset or first recognition during pregnancy [2]. A woman can also be diabetic prior to pregnancy and that falls into two categories: type 1and type 2. Type 1 diabetes occurs due to a lack of pancreatic islet beta cells, caused by autoimmune destruction and resulting in an absence of insulin; while type 2 diabetes occurs due to insulin resistance and beta cell dysfunction and is likely to be the result of interactions between genetic, environmental and immunological factors including diet, physical activity and obesity [3]. Women diagnosed with diabetes prior to pregnancy (pre-existing diabetes) will experience an increase in insulin demands during pregnancy [4].

Diabetes can have significant impacts on maternal, fetal and neonatal outcomes. The presence of diabetes can increase the risk of stillbirth by five times, and the risk of neonatal death by three times [5]. Studies have shown perinatal mortality rates are two to three times higher amongst babies of diabetic women as opposed to the general population. Also higher rates of congenital anomalies in babies of women with diabetes have been reported compared to the general population [6,7]. Since the introduction of insulin as a treatment for diabetes, mortality and morbidity rates have improved; still, they remain significantly higher than those of the general population. Longer term glycaemic control in women with diabetes is critical to satisfactory pregnancy outcome.

In order to decrease the rate of stillbirths among maternal diabetics, a variety of interventions have been adopted. The keystone of these interventions has been early detection of diabetes with monitoring of blood glucose levels and where needed interventions to ‘normalize’ these so that complications can be reduced. The objective of this review is to assess the impact of the detection and control of maternal diabetes on stillbirths using data from randomized studies, if available, or from observational studies.

## Methods

### Literature search

In order to collect evidence for the effect of detection and control of diabetes during pregnancy on stillbirths or perinatal mortality as outcomes, we conducted a systematic search of all the literature published till July 2010. The databases searched were PubMed, Cochrane Database of Systematic Reviews and WHO Regional Databases. Besides, hand search of bibliographies of relevant reviews was performed. Experts in the field were contacted for further data or for unpublished trials. The following search strategy was utilized for PubMed:

("Diabetes Mellitus"[Mesh] OR "Diabetes Mellitus, Type 2"[Mesh] OR "Diabetes Mellitus, Type 1"[Mesh] OR "Glucose Intolerance"[Mesh] OR "Diabetes, Gestational"[Mesh] OR "diabetes*" OR "gestational diabetes" OR "glucose intolerance") AND ("Pregnancy"[Mesh] OR "Pregnancy Trimester, Third"[Mesh] OR "Pregnancy Trimester, Second"[Mesh] OR "Pregnancy Trimester, First"[Mesh] OR "Pregnancy Trimesters"[Mesh] OR "pregnan*" OR "mother*" OR "maternal" OR "Mothers"[Mesh]) AND ("Fetal Mortality"[Mesh] OR "Stillbirth"[Mesh] OR "Perinatal Mortality"[Mesh] OR "Fetal Death"[Mesh] OR "Embryo Loss"[Mesh] OR "Fetal Viability"[Mesh] OR stillbirth* OR "fetal death*" OR "Fetal loss" OR "perinatal mortality" OR miscarriage* OR abortion* OR "baby’s death" OR "death of baby" OR "infant death" OR "death of fetus" OR "intrauterine death*")

For the purpose of our review, stillbirth was defined as the expulsion of a dead fetus from the body of the mother after 28 weeks of gestation. Perinatal mortality was defined as stillbirths plus the deaths within the first seven days of birth.

### Inclusion/exclusion criteria

#### Inclusion criteria

•	All studies linking screening, control and detection of diabetes during pregnancy with stillbirth (or perinatal mortality) were included

•	The studies selected were from both developed and developing countries.

•	All studies were included irrespective of language. For non-English articles, we primarily relied on the abstracts but did not translate the entire article into English.

•	All studies were included in the meta-analyses irrespective of the methodological quality of the study.

•	Studies were included in our review if they reported on interventions for diabetic pregnancies and had data on stillbirth or perinatal mortality as outcomes.

#### Exclusion criteria

•	Studies were excluded if they did not report on relevant outcomes i.e. stillbirth or perinatal mortality.

•	Studies were excluded if they did not focus on the selected interventions (screening/monitoring/pharmacotherapy).

The studies were grouped according to the following interventions: (1) impact of early detection (1^st^ trimester) strategies versus late detection (24^th^ to 28^th^ week of gestation i.e. regular screening ) of gestational diabetes mellitus (GDM) in pregnancy; (2) outpatient versus hospital based monitoring and management; (3) intensified management (dietary advice, increased frequency of monitoring, increased clinic visits or pharmacotherapy) versus conventional management strategies for diabetes in pregnancy; (4) Optimal versus suboptimal glucose control; (5) pre-conception care versus no/conventional care (6) effectiveness of continuous infused insulin versus multiple dose insulin; (7) comparisons between different types of insulin; (8) oral hypoglycemic agents and metformin use in diabetic pregnancies versus insulin treatment or no treatment; (9) Pregestational and gestational DM versus normal pregnancy.

### Data abstraction

The data were extracted by two researchers into a standard Web excel sheet prepared by the CHERG/LiST review group [8]. The variables considered included, for example, location of the study, setting, study design, blinding assessment, allocation concealment, intention-to-treat analysis, lost to follow-up rates, intervention and control group definitions and study limitations.

### Study characteristics and quantitative data synthesis

The study designs considered were randomized, quasi-randomized trials and observational studies. We generated meta-analyses using RevMan 5.0 software for outcomes where more than one study was available [9]. The assessment of statistical heterogeneity among trials was done by visual inspection i.e. the overlap of the confidence intervals among the studies, and by the Chi square (P-value) of heterogeneity in the meta-analyses. A low P value (less than 0.10) or a large chi-squared statistic relative to its degree of freedom was considered as providing evidence of heterogeneity. The I^2^ values were also looked into, and an I^2^ value greater than 50% was taken to represent substantial and high heterogeneity. In situations of substantial or high heterogeneity being present, causes were explored, sub-group analyses performed and random effects model was used; and although, this random model is not a substitute for a thorough investigation of heterogeneity, it was primarily to take into account heterogeneity that could not be explained.

Summary estimates were presented as relative risk with 95% confidence interval. For cluster randomized trials, the cluster-adjusted values were used if reported in the studies themselves. We also applied the CHERG adaptation of the GRADE criteria to grade the evidence presented by the studies in our meta-analyses [8,10]. The Child Health Epidemiology Reference Group (CHERG) guidelines are applied by scientists conducting reviews of intervention effects for use in The Lives Saved Tool (*LiST*). The six steps in the CHERG intervention review process include: (i) defining the scope of the review; (ii) conducting the literature search; (iii) extracting information from individual studies; (iv) assessing and summarizing the evidence; (v) translating the evidence into estimates of intervention effects and (vi) presenting the results.

The Lives Saved Tool (LiST) uses estimates of the effects of interventions on cause-specific child mortality as a basis for generating projections of child lives that could be saved by increasing coverage of effective interventions. Estimates of intervention effects are an essential element of LiST, and need to reflect the best available scientific evidence[8].

The World Bank list of economies (July 2009) [11] was used to classify countries into developing and developed. Low- and middle-income countries were taken as developing, while high income countries were taken as developed.

### Delphi process for establishing expert consensus

For the intervention of diabetes screening and management, in general, during pregnancy, we also sought expert consensus via the Delphi method. The Delphi technique (subsequently referred to as the Delphi) uses a series of sequential questionnaires or ‘rounds’, interspersed by controlled feedback, that seek to gain the most reliable consensus of opinion among a group of experts. It is a technique that is useful for situations where individual judgments must be tapped and combined in order to address a lack of agreement or incomplete state of knowledge. As such, the Delphi is particularly valued for its ability to structure and organize group communication [12]. The panel invited to participate were experts in maternal health representing six WHO regions (South Asia, Africa, Western Europe, Eastern Europe, North America, Australia), and including multiple disciplines - international health, obstetrics/gynecology, midwifery, etc. Thirty-one experts agreed to participate in the Delphi process. The questionnaire was developed by MYY and ZAB, and refined after several rounds of pilot testing. The questionnaire was sent by email. It included the background and aims of the Delphi and estimates of effect that were available from the literature for different scenarios. The median response and range were determined for each question. Consensus was defined a priori as an inter-quartile range in responses of not more than 30% for each question. For those estimates not reaching consensus, the plan was for results to be electronically distributed to the panel, virtual discussion allowed, and a second round of email questionnaires sent. However, consensus was achieved after one round of questionnaires and subsequent rounds were not necessary.

## Results

**Figure 1:**
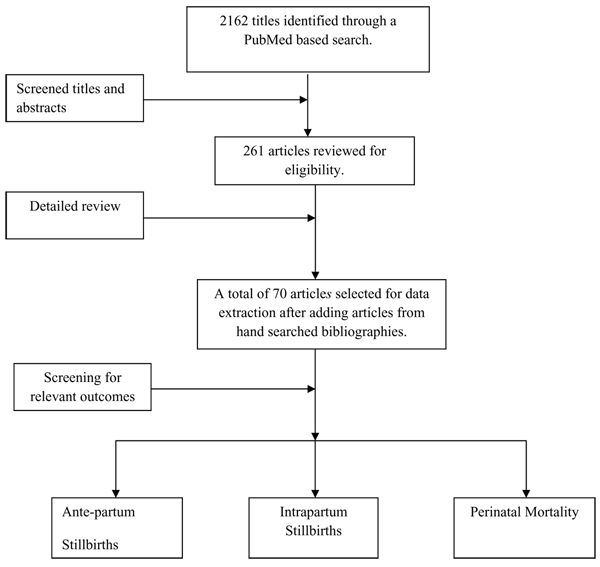
Synthesis of literature search in this review of the effect of detection and management of maternal diabetes mellitus on perinatal mortality as an outcome of pregnancy.

The above mentioned search strategy yielded 2162 hits that were reviewed and 261 abstracts of interest were preliminarily selected. After careful review of these studies, 70 studies were reviewed in detail. A flow diagram detailing the study abstraction process is given below (figure 1). Our search yielded 14 intervention studies [13-25] and 56 observational studies [16,26-80]. The studies included in review articles, which were not in our short-listed articles, were added as hand searched from bibliography. In depth characteristics of these studies are presented in Additional File 1.

### (1) a. Impact of early detection strategies versus late detection of GDM in pregnancy

A single cohort study [38] compared early testing (before 24 weeks) versus regular schedule (i.e. glucose challenge test/OGTT between 24th to 28^th^ week of gestation) of testing in a group of women with a history of GDM in prior pregnancy. This study was unable to show that early testing was better than late testing in improving perinatal mortality. This is supported by a review by Hillier et al. [81] that also did not find any randomized trials evaluating risks or benefits of early detection.

### (1) b. Other screening strategies

Two other observational studies focused on screening of diabetes using different criteria. The cohort study by Schmidt et al 2001 [68] compared two sets of screening criteria for GDM; ADA (American Diabetic Association criteria) versus WHO criteria. There were a total of 13 deaths (7 fetal deaths and 6 early neonatal deaths) with no difference using either set of criteria (ADA criteria: RR 3.1,95 CI% 1.42-6.47; WHO criteria: RR 1.59, 95% CI 0.86-2.90). A study by Ezimokhai et al 2006 [40] compared effect of universal screening (2001-2002) versus selective screening (1996-1997) of high risk candidates. No significant difference in intrauterine fetal death was observed (RR 0.42, 95% CI 0.16 -1.12) between either groups.

### (2) Outpatient versus hospital based monitoring and management

Two observational studies focusing on hospital based versus outpatient monitoring were retrieved in our search. A controlled prospective trial by Nachum et al. [62] on pregestational and gestational diabetics compared ambulatory monitoring versus repeat hospitalizations. No difference in effect on risk of perinatal mortality or neonatal mortality was observed (RR 0.98, 95% CI 0.14-6.77). A retrospective cohort study by Traub et al.1987 [72] compared treatment in peripheral maternity units with hospital based (centralized) treatment. No difference in effect on rates of stillbirth were noted (RR=0.42, 95% CI 0.09-2.01).

### (3) Intensified management (dietary advice, monitoring or pharmacotherapy) versus no or conventional management strategies for diabetes in pregnancy

A number of studies have focused on the impact of monitoring and treatment of diabetes in pregnancy versus no care/conventional care. In our review one study [15] studied the impact of intensive monitoring versus routine monitoring of serum glucose levels in a population of pregnant women with impaired glucose tolerance. The intervention group received dietary advice, capillary blood glucose monitoring five times a week and HbA1c measurements monthly, wheras the routine monitoring group received dietary advice and monthly HbA1c measurement. No stillbirths were reported in either comparison group. This was the only study which assessed the effect of monitoring of glucose levels in women with impaired glucose tolerance in pregnancy.

**Figure 2:**
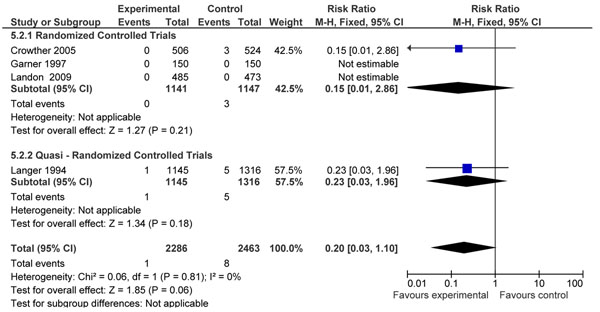
Intensive care for gestational diabetes (dietary advice, clinical monitoring +/- treatment) versus conventional management. Outcome: Pooled data for stillbirth

Four intervention studies [13,23-25] with randomized design assessed the same comparison of intensified versus conventional management in women with GDM. Pooling of results from these three studies displays an 80% non-significant reduction in risk of stillbirth (RR=0.20, 95% CI: 0.03- 1.10) when intensive management protocols are utilized (figure 2). Of these studies, Langer et al. 1994 focused on assessing the effect of intensified monitoring (7 checks of serum glucose/day) versus conventional monitoring in a group of women with gestational diabetes. This was coupled with treatment as dictated by serum glucose levels. A non significant effect was seen on risk of stillbirths (RR=0.23, 95% CI 0.03-1.97) and neonatal mortality (RR=1.49, 95% CI 0.23- 5.68). A randomized controlled trial by Crowther et al. 2005 consisting of 1031 subjects, reported a non-significant impact on the risk of perinatal mortality (RR = 0.09; 95% CI: 0.01 – 1.70) when gestational diabetics received individualized dietary advice, serum glucose monitoring and insulin therapy compared to routine care. Three stillbirths were reported in the routine care group (p-value 0.25). A similar trial by Garner et al 1997 conducted on patients with GDM comprising a small sample size (300 subjects) did not report any stillbirths. An RCT conducted by Landon et al. 2009 [25] focused on intensive treatment of gestational diabetes versus conventional management. It did not report any perinatal deaths in either groups but displayed positive effect of intervention on risk of large for gestational age (LGA), shoulder dystocia, need for cesarean section, gestational hypertension and pre eclampsia. Table 1 gives the quality grading of the outcome(s) according to the CHERG rules.

**Table 1:** Quality grading of outcomes according to the CHERG approach using adapted GRADE criteria

No of studies (ref)	Design	Limitations	Consistency	Generalizability to population of interest	Generalizability to intervention of interest	Intervention	Control	Relative Risk (95% CI)
***Intensified versus conventional management (Stillbirths): MODERATE* outcome specific quality**								

4	3 RCTs,1 quasi RCT	Small study size in 1 study, 1 study not strictly randomized and 80% of the subjects were Hispanic so may not be generalizable.	2/4 studies showing direction of benefit, while the remaining two had zero total events	All in developed countries	Cannot separate diet controlled from diet and insulin controlled diabetes during pregnancy	1	8	RR (fixed) = 0.20 (0.03-1.10)

Observational studies which looked at similar comparisons were also identified [28,32,33,35,49,50,75]. A cohort study by Huddle et al. (2005) [50] focused on pregestational and gestational diabetes in a population of black women. Initial hospitalization, intensive monitoring and management in one group were compared to only two weeks of intensive management in the group that enrolled late. Positive effect was seen on rates of stillbirth in patients with GDM and pregestational diabetes [Stillbirth: GDM (RR=0.23, 95%CI 0.12-0.47), type 1 diabetes (RR=0.32, 95%CI 0.15-0.72), type 2 diabetes (RR=0.077, 95%CI 0.019-0.32)].

Three observational studies assessed women for diabetes during pregnancy followed by treatment for those who tested positive. Those who were tested positive and treated were the compared to those that tested negative. [27,54,64] A retrospective cohort by Koukkou et al. 1995 [54] tested high risk women for GDM, followed by comparison of outcomes between women who tested positive with those that did not. No significant impact on stillbirth rates was seen (RR 2.08, 95% CI 0.19-22.7). Similar results were seen in a cohort study by Philipson et al 1985 [64] who compared women who tested positive for GDM versus those who did not. A study by Beischer et al compared women who tested positive for GDM versus those that were normal. This study screened the population for gestational diabetes and assessed effect on perinatal mortality. The study also gave data on difference in mild and severe gestational diabetes. It reported a decreasing trend in stillbirth rates between 1971 and 1994.

Applying CHERG Rules for Evidence Reviews for LiST, we gave a ‘moderate’ level quality evidence for the impact of intensified versus conventional management on stillbirths [8]. The total number of events was less than 50 (Rule 0 applies) and so there is insufficient evidence to recommend this estimate for the LiST model. One of the studies was quasi randomized [13] and another [24] had a small sample size from which conclusions could not be drawn. We, therefore, use the Delphi results to make a recommendation for the intervention of diabetes screening and management.

### (4) Optimal versus suboptimal glucose control

**Figure 3:**
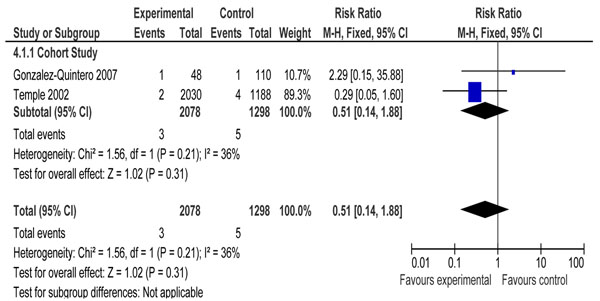
Patients with optimal control versus suboptimal control. Outcome: Pooled data for stillbirth.

**Figure 4:**
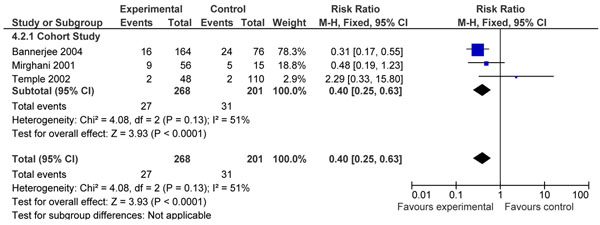
Patients with optimal control versus suboptimal control. Outcome: Pooled data for perinatal death.

Four observational studies [26,42,61,70] focused on the comparison of optimal control of serum glucose versus suboptimal control. Two studies [42,70] reported data on stillbirths and three [26,61,70] had data on perinatal mortality. Pooled data from these studies did not display a significant difference in risk of stillbirth (RR=0.51, 95% CI 0.14-1.88) (figure 3). However, perinatal mortality was significantly reduced (RR=0.40, 95% CI 0.25- 0.63) (figure 4).

For the comparison of optimal versus suboptimal control of diabetes two cohort studies [42,70] reporting stillbirth risk were identified. Of these only one study [70] had adjusted for confounding factors. The outcome of stillbirth was given ‘low’ evidence because these were cohort studies and could not be used for recommending into the LiST tool because the total events were less than 50 (Rule 0 application) [8].

### (5) Pre-conception care for pregestational diabetes versus No care/conventional care

Four observational studies [29,36,71,74] focused on the impact of preconception care (information about need for optimization of glycemic control before pregnancy, assessment of diabetes complications, review of dietary habits, intensification of capillary blood glucose self-monitoring and optimization of insulin therapy) versus no preconception care in pre gestational diabetes. Two of these studies included both type 1 and 2 diabetics [29,74] and the other two focused on type 1 diabetes only [36,71]. Of these 3 studies, one [74] was not adjusted for confounding factors and one was a multicentre cross sectional study; the evidence from these trials was deemed to be of ‘moderate’ outcome specific quality. Two studies (Temple et al.2006 [71] and Boulot et al. 2003 [29]) focused on providing intensive preconception care including information about need for optimization of glycemic control before pregnancy, assessment of diabetes complications, review of dietary habits, intensification of capillary blood glucose self-monitoring and optimization of insulin therapy. One study [74] focused only on preconception counseling. The study by Cyganek et al.2010 [36] focused on pre pregnancy planning in women who were receiving continuous subcutaneous insulin infusion or multiple daily injections. There were 14 malformations, stillbirths, and perinatal infant deaths in the not-planning versus five in the planning group (P = 0.07).

### (6) The effectiveness of continuous infused insulin versus multiple dose insulin

Seven studies focused on the comparison of continuous subcutaneous insulin versus conventional multiple injections of insulin. Six of these studies were intervention studies [16-21] and one was an observational study [47].

Of the six intervention studies [16-21] five were on type 1 diabetics and one included both type 1 and 2 diabetics [20]. Data from one study could not be added as the full text of the article was unavailable[17].

The single observational study retrieved for this comparison did not report any significant effects on perinatal mortality (p-value = not significant).

**Figure 5:**
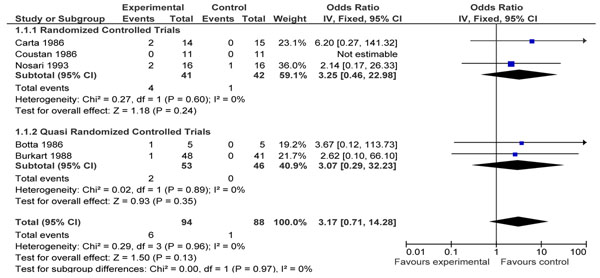
Continuous SC insuline pump versus conventional mulitiple insuline injections. Outcome: pooled stillbirth/IUFD.

Data for stillbirths from the intervention studies was pooled and the following non-significant effect was seen (OR= 3.17, 95% CI 0.71-14.28) (figure 5).

In conclusion, three RCTs [16,20,21] and 2 quasi RCTs [18,19] focused on the intervention of continuous subcutaneous insulin infusion versus conventional multiple insulin injections. None reported significant results. The meta-analysis did not reveal statistically significant impact on stillbirth risk. Studies provided a moderate level of evidence for showing no significant benefit of continuous subcutaneous insulin compared to multiple dose therapy on risk of stillbirths.

### (7) Comparisons between different types of insulin

Four studies; two RCTs [14], [22] one retrospective cohort [78] and one prospective cohort study [63] on compared different types of insulin. Of the two randomized trials, Hod et al. 2008 [14] compared the effect of insulin aspart versus human insulin. The comparison of insulin aspart versus human insulin on stillbirths did not show significant effect on the risk of stillbirth (RR=1.05, 95%CI 0.07-16.7).However, there were only 322 subjects in this trial. Persson et al. 2002[22] studied the effects of insulin Lispro versus regular insulin. There were no stillbirths or neonatal deaths reported in this comparison. A single observational study by Aydin et al.2008 [78] assessed the comparison of insulin lispro versus regular human insulin and did not report a significant difference in on the risk of stillbirth (RR=2.14, 95% CI 0.14-33.03). Whereas the other prospective cohort[63]compared the effects of glargine versus NPH insulin. There were two stillbirths reported in the group of pregestational diabetics using NPH insulin and none in the glargine utilizing group (p-value=0.028).

### (8) Oral hypoglycemic agents and metformin use in diabetic pregnancies

Five observational studies [39,46,48,51,65] focused on the impact of using oral hypoglycemic agents or metformin versus diet alone or insulin. Of these studies Holt et al. 2008 [48] compared the effect of glibenclamide versus insulin in a population of women with GDM. No significant difference was seen on risk of stillbirth (RR= 3.07, 95%CI 0.13-73.31). A retrospective cohort study by Ekpebegh et al 2007 [39]compared women treated with oral glucose-lowering agents (OGLA-metformin and glibenclamide) alone (group 1) and women converted from OGLA to insulin (group 2) with women who were treated with insulin alone and women converted from diet to insulin (group 3). There was a significant difference in perinatal mortality rates between group the three groups; perinatal mortality rates (per 1000 births): 125, 28 and 33 (p-value=0.003). At least one group differed significantly from another (p-value=0.003). It is clear that the PNM rate for those on OGLA alone, 125 per 1000, was much higher than for the other two groups. The majority of all perinatal deaths were stillbirths: eight of 11 in the OGLA alone group and five of seven in the OGLA to insulin group.

A study by Hughes et al 2006[51] compared use of metformin versus none in type 2 diabetics. No significant difference was observed in perinatal loss (p-value =0.65). Another retrospective cohort study Rayburn et al 2006 [65] compared pregnancies requiring oral hypoglycemic drugs with those controlled with diet alone. A single stillbirth was reported in the diet controlled group with none in the treatment group and the results were not significant. Hellmuth et al. 2007 compared metformin with sulphonylureas in pregnant diabetic patients and a reference group treated with insulin.

### (9) Pregestational and gestational DM versus normal pregnancy

Our literature search also yielded studies which assessed the risk of stillbirth in the above mentioned comparison. Twelve observational studies assessing the risk of stillbirth in pre-gestational and gestational diabetes versus normal pregnancies [82-93] were identified. Of these, five studies [82-86] could be pooled to assess risk of stillbirth in women with diabetic pregnancy versus normal women. Pooled data from these studies displayed a significant effect on stillbirth risk in the fixed effects model (RR 2.17, 95% CI 1.45-3.25). Due to considerable heterogeneity I^2^=95% and p-value <0.10, a random effects model was also applied which indicated that the results were statistically insignificant (RR=3.38, 95%CI 0.49-23.34).

### Delphi results

**Figure 6:**
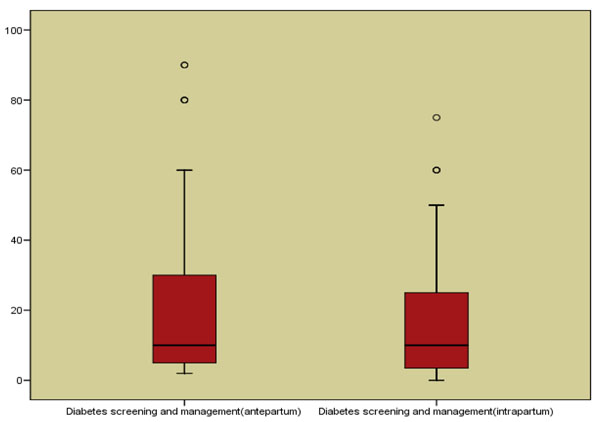
Box plots for the Delphi results on diabetes screening and management during pregnancy.

Figure 6 summarizes the estimates (medians) from the Delphi consensus for the effect of intervention of diabetes screening and management versus no specific identification or care for women with diabetes. The experts determined the effect to be 10% reduction in antepartum stillbirth (or intrauterine death). (interquartile range of 5% to 30%) and 10% reduction in intrapartum stillbirth (interquartile range of 3.5% to 25%). These figures have been recommended for use in the LiST model.

## Discussion

This systematic review attempts to summarize the evidence in the literature regarding the prevention and management of gestational and pregestational diabetes and the effect on stillbirths. We have performed meta- analyses where possible to estimate impact of prevention and treatment strategies on stillbirth. We found substantial differences in clinical and methodological approaches across all studies with variability in interventions, definitions of outcomes, study design and risk of bias.

Although the focus was on interventions having an impact on stillbirth risk we found that there was a lack of data regarding this outcome in most studies. Some studies included present stillbirths as part of perinatal mortality. With regard to screening strategies no randomized controlled trial has been reported that directly evaluates the risks and benefits of early diabetes screening [81]. Our systematic review identified 4 studies focusing on intensive treatment of diabetes versus conventional management. A reduction of 80% was observed in stillbirth risk with intensive management but was not statistically significant. A systematic review by Alwan et al. 2009[4] focusing on treatment of gestational diabetes reported positive benefits on maternal preeclampsia (five trials, 1255 women; RR = 0.95, 95% CI 0.80 to 1.12) and macrosomia when any specific treatment is utilized versus routine antenatal care for GDM, however, results were not significant. A recent review by Horvath et al. 2010 [94] focusing on the same comparison reported similar effects on risk of macrosomia, shoulder dystocia and preeclampsia.

For the comparison of optimal versus suboptimal control of diabetes two cohort studies [42,70] reporting stillbirth risk were identified. Three studies [26,61,70] reported perinatal mortality for the same comparison; and a 60% reduction in risk of perinatal mortality was seen in women with optimal glucose control, which was statistically significant. A similar comparison was performed by Inkster and colleagues 2006 [5]. Similar findings were reported in their review regarding perinatal mortality. Increased perinatal mortality was associated with poor glycaemic control, pooled odds ratio 3.03 (95% CI: 1.87 to 4.92).

For the intervention of continuous subcutaneous insulin infusion versus conventional multiple insulin injections a systematic review by Mukhopadhay et al. 2007 [95] was identified. This review also reported that no significant difference in glycemic controls or pregnancy outcomes including stillbirths was seen when the two different treatment strategies (continuous subcutaneous insulin versus conventional daily injections) were compared. It is noted that studies included in the pooled analyses had small sample sizes and further studies with larger sample sizes may be needed for more conclusive results [95].

In the treatment of diabetes during pregnancy the spotlight is often on insulin therapy; however, in recent years studies have been done that assess the impact of oral hypoglycemic agents and metformin on pregnancy outcomes. A recent systematic review concluded that no significant differences were found in maternal glycemic control or cesarean delivery rates between the insulin and glyburide groups. There was a higher proportion of infants with neonatal hypoglycemia in the insulin group (8.1%) compared with the metformin group (3.3%) (P-value: 0.008). Also the rate of congenital malformations, which contribute to stillbirth risk, did not differ between pregnancies treated with insulin and those treated with oral agents [96]. Another systematic review [4] reported a 54% reduction in the rates of cesarean section in women who received oral hypoglycemic therapy compared to insulin. Currently, data are insufficient to conclude that the use of oral hypoglycemic agents reduces the risk of stillbirth in women who have GDM.

Diabetes in pregnancy is also associated with higher rates of miscarriage, pre-eclampsia, preterm labour and higher rates of fetal malformation [97]; neural tube defect, urinary tract disorder, macrosomia, birth injury, and perinatal mortality. These risks can be minimized by optimal glycaemic control, both prior to and throughout the pregnancy [98,99], and this is best achieved through comprehensive preconception care where other issues such as genetic risks, health status, reproductive history, exposure to environment toxins, immunization and life-style risk factors can be addressed via a community based approach to manage diabetes before and during pregnancy [100].

## Conclusions

Diabetes, and especially pregestational diabetes with its attendant vascular complications, is clearly a significant risk factor for stillbirth. Our review highlights the fact that very few studies of adequate quality are available that can provide data on the impact of screening and management of diabetes in pregnancy and stillbirth risk. Applying the CHERG rules, we deduced a 62% reduction in stillbirths due to intensive vs. conventional management of gestational diabetes. The Delphi process however, suggested a more conservative figure of 10% reduction in the risk of antepartum and intraprtum stillbirths and presently these point estimates are being recommended for use in LiST.

## Competing interests

The authors declare no conflicts of interest.

## Authors’ contributions

Professor Zulfiqar A Bhutta developed the review parameters and secured support. Dr. Madiha Syed, Hasan Jawed and Dr. Mohammad Yawar Yakoob undertook the literature search, data extraction and analysis under the supervision of Professor Bhutta. Dr. Zulfiqar A. Bhutta gave advice in all the aspects of the project and was the overall supervisor.

## Supplementary Material

Additional file 1 This paper has an additional file that contains all of the studies that were graded for quality for inclusion in the analyses. This file is a word document Click here for file
