# Supplementary material for: Effect of screening and management of diabetes during pregnancy on stillbirths
Source: BMC Public Health. 2011 Apr 13;11(Suppl 3):S2. doi: 10.1186/1471-2458-11-S3-S2 (PMC3231893; doi:10.1186/1471-2458-11-S3-S2)
Supplement: Additional file 1 — This paper has an additional file that contains all of the studies that were graded for quality for inclusion in the analyses. This file is a word document [file 1471-2458-11-S3-S2-S1.docx]

**Additional File 1:** Characteristics of included studies

| **Study, Year (Reference)** | **Design** | **Country** | **Patients, n** | **Population characteristics** | **Intervention components** | **Timing** | **Control** |
| --- | --- | --- | --- | --- | --- | --- | --- |
| **Developing countries** |  |  |  |  |  |  |  |
| Akhter 1996 [84] | Retrospective study | Pakistan | 6380 | Pregnant women undergoing either a 75 g glucose tolerance test or a screening 50 g glucose challenge. | Diabetic pregnancies | Pregnancy | Non-diabetic pregnancies |
| Al-Dabbous 1996[1] | Prospective cohort study | Saudi Arabia | 133 | Pregnant diabetic women. Maternal diabetes mellitus diagnosis was based on WHO criteria | Women on insulin therapy during pregnancy. | Pregnancy | Women who were either not being treated with insulin or not booked for antenatal care and level of management of their diabetes was not available |
| Banerjee 2004[29] | Comparative study | India | 240 | Mothers with diabetes mellitus (DM) and pregnancy. These women were put on exercise, diet and or insulin therapy. Glycaemic parameters monitored include fasting plasma glucose (FPG), 2 hr. postprandial plasma glucose (PPPG) and HbA1C. | Women with Tight glycemic control (FPG < 70 mg/dl, PPPG < 100 mg/dl, HBA1C < 6.5%) | Pregnancy | Women with acceptable control (FPG 70-95 mg/dl, 2 hr. PPPG 100-120, HBA1C 6.5-7.5% and UC had FPG > 95 mg/ dl, 2 hr. PPPG > 120 mg/dl and HBA1C > 7.5%). |
| Bassaw 1995[89] | Retrospective study | Trinidad | 260 | Pregnant diabetic patients. | No intervention association between glycemic control and perinatal outcomes was assessed . | Pregnancy | No intervention association between glycemic control and perinatal outcomes was assessed . |
| Coetzee 1985[37] | Full text not available to assess study design | South Africa | 503 | Non-insulin-dependent pregnant diabetics (NIDD), using appropriately constituted calorie-restricted diets with the oral agents metformin and glibenclamide as may be necessary, with rapid recourse to insulin if the latter do not produce excellent control of blood glucose. | New diabetics (ND, diagnosed during pregnancy)-group 1, Established diabetics (known diabetics, KD)-group2 | Pregnancy | Untreated women. |
| Coetzee 1980[36] | Observational dstudy | South Africa | 171 | Pregnant women with established insulin-independent diabetes. | Pregnant women managed primarily by regulating diet; when this failed metformin or glibenclamide therapy was instituted. Insulin was used when diet and oral drugs failed. | Pregnancy | Untreated group |
| Coetzee 1979[35] | Comparative study | South Africa | 127 | Women with gestational diabetes mellitus. | Women treated principally by regulating diet, but when this failed metformin or glibenclamide therapy was instituted. Insulin was used when diet and oral drugs failed. | Pregnacy | Untreated group |
| Daponte 1999[40] | Retrospective cohort study | South Africa | 170 | Diabetic pregnant women. | Women treated with established insulin regimen. | Pregnancy | Control women not treated with the established insulin regimen. |
| Ekpebegh 2007 [42] | Retrospective cohort study | South Africa | 379 | Pregnant women with Type 2 diabetes mellitus (singleton pregnancies > or = 24 weeks). | Women treated with oral glucose-lowering agents (OGLA-metformin and glibenclamide) alone (group 1) and women converted from OGLA to insulin (group2). | Pregnancy | Women treated with insulin alone or converted from diet alone to insulin (group3). |
| Ezimokhai 2006 [43] | Audit | United Arab Emirates | 1495 | Pregnancies complicated by diabetes mellitus (DM) in a multiethnic population. | Diabetic pregnancies detected by universal screening between the years 2001-2002. | Pregnancy | Diabetic pregnancies detected by selective screening between the years 1996-1997 |
| Fraser 1982[91] | Review of records | Kenya | 55 | Pregnant women | Pregnancies complicated by diabetes | Not applicable | Nondiabetic population |
| Kadiki 1993 [56] | Audit | Libya | 988 | Pregnant diabetic patients, twelve patients were insulin-dependent (type 1) and 976 patients were non-insulin-dependent (type 2 | Audit was done to see various outcomes |  | Audit was done to see various outcomes |
| Huddle 1993 [52] | Cohort study | South Africa | 500 | Pregnant diabetic women of African ethnic origin from Soweto. | A total of 354 pregnancies (147 gestational, 207 pregestational), managed over an 8 1/2 year period. | Pregnancy | Control group |
| Huddle 2005 [53] | Cohort Study | South Africa | 941 | Diabetic pregnant black women including gestational diabetes mellitus and pregestational (type 1 and type2) diabetes mellitus | Women initially hospitalized for clinical and biochemical assessment, ultrasonographic examination and determination of treatment. Patients were taught home blood glucose monitoring. Patients returned fortnightly for follow-ups till 32 weeks' gestation and then weekly till 37 weeks' when they were readmitted till delivery. | Pregnancy | Women who, because of late referral or presentation, were unable to receive more than 2 weeks of intensive management before delivery. Delivery was performed as soon as fetal maturity was confirmed. |
| Lutale 1991 [60] | Audit | Tanzania | 47 | Pregnant diabetic Tanzanian women. | Audit was done to see various outcomes |  | Audit was done to see various outcomes |
| Mirghani 2000 [64] | Prospective cohort study | Sudan | 71 | Women diagnosed with diabetes mellitus going through pregnancy. Women with histories of stillbirth or early neonatal death, or baby weighing above 4.5kg or more, or diabetes in 1st degree relatives, or congenital malformed babies, or glycosuria, or symptoms underwent a glucose tolerance test. | Patients with good(group 1) or satisfactory(group 2) glycemic control. | Pregnancy | Patients with poor glycemic control. |
| Mogokong 1983 [90] | Clinical trial | South Africa | 48 | Pregnant Black patients | Pregnant Black patients with overt diabetes treated with a protocol of strict control of plasma glucose levels.  Subcutaneous insulin therapy was used if the above levels were persistently exceeded | Pregnancy | General hospital population(normal pregnancies) |
| Schmidt 2001 [71] | Cohort study | Brazil | 4977 | Women greater than or equal to 20 years of age, between 20th and 28th gestational week with a negative history of diabetes outside of a pregnancy. | Women identified as gestational diabetics using American Diabetes Association(ADA) criteria following a 2-h 75-g oral glucose tolerance test between their 24th and 28th gestational week. | Pregnancy | Women identified as gestational diabetics based on the WHO criteria following a 2-h 75-g oral glucose tolerance test between their 24th and 28th gestational week. |
| Sobande 2005[72] | Retrospective cohort study | Saudi Arabia | 185 | Pregnancies complicated by diabetes mellitus. | Type 1- insulin dependent diabetics (group 1), type 2- non insulin dependent diabetic patients (group 2) | Pregnancy | Gestational diabetic patients (group 3) |
| **Developed countries** |  |  |  |  |  |  |  |
| Artner 1981 [78] | Observational Study | Austria | 316 | Pregnant diabetic women. | Women with Prognostically Bad Signs of Pregnancy. | Pregnancy | Overall pregnant diabetic women visiting the hospital. |
| Aucott 1994 [79] | Retrospective cohort study | USA | 156 | All prepartum gravid patients. | Prepartum gravid patients with insulin dependent diabetes mellitus receiving rigorous management during pregnancy(glucose levels monitored 4 times daily and maintained by either continuous insulin infusion pumps or split -dose therapy) | Pregnancy | Prepartum gravid patients receiving normal treatment. |
| Ayromlooi [81]1977 | Comparative study | Full text not available | Full text not available | Pregnant diabetic patient. | Modern antepartum and intrapartum fetal monitoring technics used in management. | Pregnancy | Conventional management |
| Bancroft 2000[18] | RCT | United Knigdom | 68 | Pregnant women with impaired glucose tolerance between 24 -28th week of gestation | Women receiving intensive diabetic monitoring (standard dietary advice and capillary glucose monitoring 5 times a week and monthly glycosylated haemoglobin measurements | Pregnancy | Women not receing intensive diabetic monitoring (standard dietary advice and monthly glycosylated haemoglobin measurements) |
| Balsells 1997 [82] | Comparative Study | Spain | 115 | Women with gestational diabetes mellitus. | Insulin dependent women | Pregnancy | Non-insulin dependent women |
| Beischer 1996 [30] | Retrospective Cohort | Australia | 116,303 | All pregnant women who underwent a glucose tolerance test. | Women with gestational diabetes. | Pregnancy | Women with normal glucose tolerance test. |
| Berne[31] | Clinical trial | Sweden | 119 | Women with gestational diabetes (GDM) | Insulin treatment given to women. | Pregnancy | Previous pregnancies in the same women. |
| Botta 1986[21] | Quasi Randomized Controlled trial | Italy | 10 | Pregnant women with type I diabetes mellitus | Women treated with continuous subcutaneous insulin to manage their diabetes during pregnancy | Pregnancy | Women treated with intensive conventional therapy of insulin(2-4 daily injections) to manage their diabetes during pregnancy. |
| Boulot 2003 [32] | Cross-sectional Study | France | 435 | All women with type I or type II diabetes and a single pregnancy. | Pregnant pregestational diabetic women receiving preconceptional care | Periconceptual | Pregnant pregestational diabetic women not receiving preconceptional care. |
| Bourgeois 1990[34] | Audit | USA | 109 | Type I diabetic women. | Type I diabetic women managed almost entirely as outpatients | Pregnancy | Diabetic patients receiving more conventional management. |
| Burkart 1998 [22] | Quasi Randomized control | Europe | 117 | Pregnant women with type I diabetes mellitus | Women treated with continuous subcutaneous insulin to manage their diabetes during pregnancy | Pregnancy | Women treated with intensive conventional therapy of insulin(2-4 daily injections) to manage their diabetes during pregnancy |
| Carta 1986 [23] | RCT | Europe | 29 | Pregnant women with type I diabetes mellitus | Women treated with continuous subcutaneous insulin to manage their diabetes during pregnancy | Pregnancy | Women treated with intensive conventional therapy of insulin(2-4 daily injections) to manage their diabetes during pregnancy |
| Coustan 1986 | RCT | USA | 22 | Women with insulin-dependent diabetes mellitus experiencing pregnancy. | Women treated with continuous subcutaneous insulin to manage their diabetes during pregnancy. | Pregnancy | Women treated with intensive conventional therapy of insulin(2-4 daily injections) to manage their diabetes during pregnancy. |
| Crombach 1990[38] | Before and after study | Germany | 112 | Pregnancies in women with diabetes mellitus. | Women treated with intensified insulin treatment after 1981 | Pregnancy | Women treated with conventional therapy |
| Crowther 2005 [26] | RCT | Australia | 1030 | Women with a singleton or twin pregnancy between 16 and 30 weeks’ gestation, attended antenatal clinics at the collaborating hospitals, had one or more risk factors for gestational diabetes on selective screening or a positive 50-g oral glucose challenge test | Individualized dietary advice  from a qualified dietitian, instructions on how to self-monitor and insulin therapy | Pregnancy | Women receiving routine care. |
| Cyganek 2010 [39] | Comparative study | Poland | 269 | Pregnant women with type 1 DM who were treated with either contionous SC insulin infusion or conventional multiple daily insulin injections | Pre-Pregnancy planning available | Pregnancy | No pre-pregnancy care provided |
| Dong 1993 [41] | Cohort | Australia | 1027 | Women who had gestational diabetes in their previous pregnancies managed at the Mercy Hospital for Women. | Women tested before 24 weeks' gestation in the subsequent pregnancy (Group 1). | Pregnancy | Women tested after 24 weeks' gestation in the subsequent pregnancy(Group 2) and women not tested at all in their subsequent pregnancy (Group3). |
| Dunne et al.2009[94] | Cohort | Multicentre study | 104 | We studied women with established diabetes  for > 6 months before the index  pregnancy in 2006–2007. Women were  managed according to local guidelines. Five  values of A1C at the first visit; at 12, 24, and  36 weeks; and before delivery were chosen  to represent the metabolic control. An ophthalmologist  evaluated for retinopathy. Information  regarding microalbuminuria  (30–300 mg/24 h), diabetic nephropathy  (>300 mg/24 h), hypertension (pregestational  treatment or a blood pressure  >140/90 at first antenatal visit), pregnancyinduced  hypertension (blood pressure  >140/90 x two measurements), preeclampsia  (onset of blood pressure and proteinuria  >300 mg/24 h after 20 weeks),  preterm delivery <37 weeks gestation,  large for gestational age (birth weight >4  kg), and small for gestational age (birth  weight<2.5 kg) were recorded. | Central hospital | Pregnancy | Peripheral hospital |
| Fadel 1982 [44] | Observational study | Full text not available | 107 | Pregnant women with diabetes mellitus. | Frequent prenatal visits, strict metabolic control, a program for antepartum fetal surveillance using estriols and contraction stress testing, liberal hospitalization, fetal lung maturity assessment, individualization of the time of induction of labor and careful intrapartum fetal monitoring. | Pregnancy | Not applicable |
| Garner 1997 [27] | RCT | Canada | 300 | All pregnant women diagnosed with gestational diabetes mellitus between 24 and 32 weeks' gestation in an otherwise low risk pregnancy. | Dietary advice and calorie restricted diet of 35kcal/kg ideal body weight per. They were also taught home glucose monitoring techniques. Women were seen biweekly and biophysical profiles were performed at each visit with ultrasonographic assessment. | Pregnancy | Women received routine obstetric care comprising of no dietary advice and performance of two weekly at home glucose level checks with whole blood glucose reagent strips. |
| Gonzalez-Quintero 2007 [45] | Retrospective cohort study | USA | 3218 | Patients with singleton gestation enrolled in an outpatient gestational diabetes mellitus management program for at least 7 days who delivered at term | Patients with suboptimal blood glucose control(women with blood glucose averages higher than the recommended guidelines). | Pregnancy | Patients with optimal blood glucose control(women with blood glucose averages within the recommended guidelines). |
| Gregory 1992 [46] | Comparative study | United Kingdom | 139 | Pregnancies in women with type 1 (insulin dependent) diabetes. | Pregnancies in women with type 1 (insulin dependent) diabetes in 1984-1990 | Pregnancy | Pregnancies in women with type 1 (insulin dependent) diabetes in 1977-1983. |
| Gyves 1977 [47] | Observational study | Full text not available | 96 | Pregnant diabetic patient. | Women managed with increased understanding of maternal-fetal carbohydrate homeostasis | Pregnancy | Women treated conventionally |
| Hanson 1993[48] | Prospective study | Sweden | 279491 | Pregnant women. | Insulin-dependent diabetic pregnancies. | Pregnancy | Normal pregnancies. |
| Hellmuth 2000 [49] | Cohort study | Denmark | 160 | Orally treated pregnant diabetic patients | Women treated with metformin during pregnancy. | Pregnancy | Women treated with sulphonylurea during pregnancy and a reference group of diabetic women treated with insulin during pregnancy. |
| Hieronimus 2005 [50] | Comparative study | France | 56 | Pregnancies in women with type 1 diabetes. | Women treated with continuous subcutaneous insulin pump . | Pregnancy | Women treated with multiple insulin injections |
| Hod 2008[17] | RCT | 18 countries | 322 | Women aged 18 years or older and with type 1 diabetes for 1 year or longer who were pregnant or were planning to become pregnant. | Women receiving Insulin Aspart for glycemic control | Pregnancy | Women receiving Human Insulin for glycemic control |
| Holt 2008 [51] | Comparative study | United Kingdom | 144 | Women with gestational diabetes mellitus. | Women treated with glibenclamide | Pregnancy | W omen treated with insulin.  Women treated with diet alone |
| Hughes 2006 [54] | Comparative Study | New Zealand | 214 | Pregnant women with Type 2 diabetes (Type 2 DM). | Women treated with metformin | Pregnancy | Women not treated with metformin. |
| Johnstone 2006 [55] | Cohort study | United Kingdom | 567 | Women diagnosed as diabetics and taking insulin before pregnancy; the gestational age at delivery was 28 weeks or greater; the pregnancy was singleton, mainly type I diabetics. | Women in 1990s (group 1), Women in 1980s (group 2) |  | Women in 1970s (group 3), women in 1960s (group 4) |
| Karmon 2009 [85] | Retrospective cohort study | Israel | 184256 | All pregnant women | Women classified as having gestational diabetes mellitus A1 (diet controlled only). Followed-up at high risk clinics, provided with nutritional counseling, and taught to evaluate postprandial and fasting glucose values twice weekly. | Pregnancy | Women without gestational diabetes mellitus A1. |
| Laatikainen 1987 [20] | RCT | Europe | 40 | Pregnant women with type I diabetes mellitus | Women treated with continuous subcutaneous insulin to manage their diabetes during pregnancy. | Pregnancy | Women treated with intensive conventional therapy of insulin(2-4 daily injections) to manage their diabetes during pregnancy. |
| Landon 2009[28] | RCT | USA |  | Women were invited to participate in this study if,  between 24 weeks 0 days and 30 weeks 6 days of  gestation, they had a blood glucose concentration  between 135 and 200 mg per deciliter (between  7.5 and 11.1 mmol per liter) 1 hour after a 50-g  glucose loading test. | Women were assigned to receive either formal  nutritional counseling and diet therapy,12  along with insulin if required (treatment group) | Pregnancy | Usual prenatal care |
| Landon 1992 [58] | Prospective cohort study | USA | 114 | Pregnant patients with insulin-dependent diabetes mellitus using a memory-based glucose reflectance meter and undergoing fetal surveillance during the third trimester. | A non reactive last non-stress test before delivery | Pregnancy | Reactive last non-stress test before delivery |
| Langer 1994 | RCT | USA | 2461 | All women with gestational diabetes mellitus diagnosed after screening for carbohydrate intolerance between the 24th and 28th week of gestation. | Intensified management of gestational diabetics (patients self-monitored blood glucose by assigned memory reflectance meters 7 times a day) | Pregnancy | Conventional management of gestational diabetics |
| Lapolla et al.2009[95] | Prospective cohort | Italy | 3465 | Pregnant women with GDM | Pregnant women with GDM | Pregnancy | Normal controls |
| Lufkin 1984[59] | Full text not available | USA | 505 | Pregnant women | Pregnant diabetic women | Pregnancy | Control group |
| Martin 1987 [61] | Before and after study | Full text not available | 613 | Diabetes complicated pregnancies | Women with pre-existing diabetes (95% of whom were insulin-dependent) | Pregnancy | Women with gestational diabetes |
| McElvy 2000 [62] | Before and After study | USA | 306 | Pregnant Type I diabetics enrolled preconceptionally or during the first trimester (up to 14 weeks) and had pregnancies continuing beyond 20 weeks gestation. | Strict glucose control was implemented and adherence assessed. Antepartum fetal surveillance was started at 32 weeks gestation. This was done in three five year periods | Pregnancy | These comprised of periods before 1973 and after cessation of funding (1993). No preconceptional care was given. |
| Mello 1997 [63] | Comparative Study | Italy | 64 | Insulin-dependent diabetic (IDDM) pregnant women, classified from White's class B to class R were included in the study | Pregnant women who joined the study before the 9th week (early control group). | Pregnancy | Pregnant women who joined the study after the 9th week (late control group). |
| Miranda 1994 [64] | Clinical trial | Spain | 158 | Pregnant women. | Pregnant women with pregestational insulin-dependent diabetes mellitus (IDDM). The women had received several daily doses of a mixture of rapid action (regular) and intermediate action insulin with the aim of keeping preprandial glucose levels lower than 95 mg/dl and postprandial glucose levels lower than 120 mg/ | Pregnancy | Control group consisting of randomly selected, normal, pregnant women who gave birth at approximately the same time. |
| Nachum 2001[65] | Controlled prospective trial | Israel | 801 | Patients with singleton diabetic pregnancy in which therapy was started before 34 gestational weeks | Group 1: Pregnant women with pregestational diabetes mellitus receiving ambulatory care to manage their pregnancy.  Group 2: Women with gestational diabetes mellitus receiving ambulatory care. | Pregnancy | Group 3: Pregnant women with pregestational diabetes mellitus with their pregnancies routinely managed by repeated hospitalizations.  Group 4: Women with gestational diabetes mellitus routinely managed by repeated hospitalizations. |
| Negrato et al.2010 [66] | Prospective cohort study | Brazil | 138 | A prospective cohort study was conducted analyzing outcomes from 56 women with pregestational and 82 with gestational diabetes treated with either insulin regimen. | Pre gestational and gestational diabetics treated with glargine | Pregnancy | Pre gestational and gestational diabetics treated with NPH |
| Nosari 1993 [24] | RCT | Full text not available | 32 | Pregnant women with type I diabetes mellitus | Women treated with continuous subcutaneous insulin to manage their diabetes during pregnancy | Pregnancy | Women treated with intensive conventional therapy of insulin(2-4 daily injections) to manage their diabetes during pregnancy |
| Persson et al.2009[93] | Prospective cohort | Sweden | 1265 296 | This was a population-based study. Data  were obtained from the Medical Birth Registry, covering _98% of all pregnancies in Sweden. A total of 5,089 type 1 diabetic pregnancies and 1,260,207 control pregnancies were included. Odds ratios (ORs) were adjusted for group differences in maternal age, parity, BMI, chronic  hypertensive disease, smoking habits, and ethnicity. | Pregnant women with type 1 DM | Pregnancy | Normal controls |
| Persson 2002 [25] | RCT | Sweden | 33 | Pregnant  women with type 1 diabetes. | Pregnant women with type 1 DM were randomised to treatment with lispro insulin. | Pregnancy | Pregnant women with type 1 DM were randomized to treatment with regular insulin |
| Philipson 1985 [67] | Cohort study | USA | 316 | Pregnant women | Pregnant women with gestational diabetes mellitus (diagnosed by a 1-h, 50-g postprandial glucose screen or a 100-g oral glucose tolerance test or a 25-g rapid intravenous glucose tolerance test). | Pregnancy | Pregnant women without gestational diabetes mellitus matched for maternal age, maternal race, maternal weight, maternal socioeconomic status and gestational age at the first obstetric visit. |
| Rayburn 2005[68] | Retrospective cohort study | USA | 280 | A-2 diabetic pregnancies requiring primarily oral hypoglycemic therapy | Gestational diabetes not controlled with diet alone (A-2) | Pregnancy | Diet-controlled diabetic (A-1) pregnancies |
| Roberts 1990 [69] | Audit | New Zealand | 1528 | Pregnancies in women with diabetes mellitus. | Women with established diabetes. | Pregnancy | Women with gestational diabetes. |
| Temple 2006[74] | Cohort study | United Knigdom | 290 | All pregnant women with type 1 diabetes. | Women receiving prepregnancy care (Women attending the center for prepregnancy care were seen every 1–3 months by the consultant physician and diabetes specialist nurse. All were reviewed by a dietitian. Women were advised to monitor capillary blood glucose four to seven times daily and aim for preprandial blood glucose 6 mmol/l, postprandial blood glucose 8 mmol/l, and a target HbA1c (A1C) 7.5% before conception) | Pregnancy | Women not receiving prepregnancy care. |
| Temple 2002[73] | Prospective cohort study | United Kingdom | 2158 | Pregnant women with type I diabetes mellitus. | Women with hemoglobin concentration > or equal to 7.5% (defined as having poor control) at booking. | Pregnancy | Women with hemoglobin concentration < 7.5% (defined as having fair control) at booking. |
| Traub 1987 [75] | Retrospective Cohort | Northern Ireland | 2231 | Pregnancies in known insulin-dependent diabetic women. | Women treated in Royal Maternity Hospital (subdivided into those entirely treated here[group1] and those who were referred[group 2]). | Pregnancy | Women treated in peripheral maternity units. |
| Vaarasmaki 2001[86] | Audit | Finland | 1442 | Singleton pregnancies | Singleton pregnancies complicated by insulin-treated diabetes. | Pregnancy | Pregnant women without diabetes. |
| Vaarasmaki 2000[87] | Prospective cohort study | Finland | 44974 | Births at a gestational age of 22 weeks or more, or a birth weight of 500g or more. | Women with type I diabetes. Women were regularly checked and given treatment to manage their diabetes. | Pregnancy | Non-diabetic controls. |
| Willhoite 1993[77] | Prospective cohort study | USA | 185 | Pregnant women with pregestational diabetes mellitus. | Women who received preconception counseling | Preconceptal | Women who did not receive preconception counseling. |
| Wood 2003[88] | Retrospective cohort and nested case-control study | Canada | 10913 | Pregnant women. | Diabetic women who had had a pregnancy. Prediabetic pregnancies (group 1), and diabetes diagnosed pregnancies(group 2). | Pregnancy | Pregnant women without diabetes. |
| **Reviews and Meta-analyses** |  |  |  |  |  |  |  |
| Inkster 2006[6] | Systematic review | United Kingdom | 13 studies identified for review | Women with type 1 and type 2 diabetes mellitus. | Poor glycaemic control for pregnant women with diabetes. | Pregnancy | Optimal glycaemic control for pregnant women with diabetes. |
| Mukhopadhay 2007[101] | Systematic Review | United Kingdom | 182 | Pregnant diabetic women | Women treated with continuous insulin infusion | Pregnancy | Women treated with conventional multiple daily insulin injections. |
